# Kinetic Characterization of a Putatively Chitin-Active LPMO Reveals a Preference for Soluble Substrates and Absence of Monooxygenase Activity

**DOI:** 10.1021/acscatal.1c03344

**Published:** 2021-09-07

**Authors:** Lukas Rieder, Dejan Petrović, Priit Väljamäe, Vincent G.H. Eijsink, Morten Sørlie

**Affiliations:** † Faculty of Chemistry, Biotechnology, and Food Sciences, Norwegian University of Life Sciences (NMBU), Ås N-1432, Norway; ‡ Institute of Molecular and Cell Biology, University of Tartu, Tartu 50090, Estonia

**Keywords:** LPMO, kinetics, peroxygenase activity, H_2_O_2_ tolerance, redox potential, oxidase activity

## Abstract

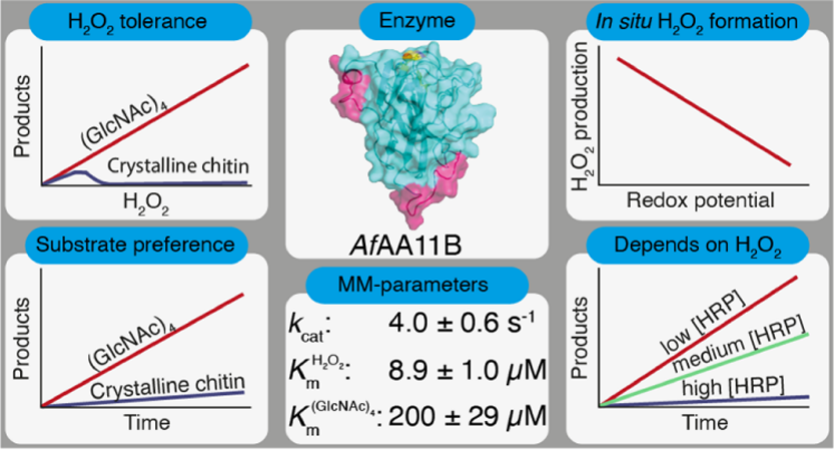

Enzymes known as lytic polysaccharide monooxygenases (LPMOs) are recognized as important contributors to aerobic enzymatic degradation of recalcitrant polysaccharides such as chitin and cellulose. LPMOs are remarkably abundant in nature, with some fungal species possessing more than 50 LPMO genes, and the biological implications of this diversity remain enigmatic. For example, chitin-active LPMOs have been encountered in biological niches where chitin conversion does not seem to take place. We have carried out an in-depth kinetic characterization of a putatively chitin-active LPMO from *Aspergillus fumigatus* (*Af*AA11B), which, as we show here, has multiple unusual properties, such as a low redox potential and high oxidase activity. Furthermore, *Af*AA11B is hardly active on chitin, while being very active on soluble oligomers of *N*-acetylglucosamine. In the presence of chitotetraose, the enzyme can withstand considerable amounts of H_2_O_2_, which it uses to efficiently and stoichiometrically convert this substrate. The unique properties of *Af*AA11B allowed experiments showing that it is a strict peroxygenase and does not catalyze a monooxygenase reaction. This study shows that nature uses LPMOs for breaking glycosidic bonds in non-polymeric substrates in reactions that depend on H_2_O_2_. The quest for the true substrates of these enzymes, possibly carbohydrates in the cell wall of the fungus or its competitors, will be of major interest.

## Introduction

Lytic polysaccharide monooxygenases (LPMOs) are receiving massive attention due to their ability to degrade recalcitrant polysaccharides, such as cellulose and chitin, in biomass conversion.^1−7^ Through the use of powerful redox chemistry, LPMOs are able to selectively activate C–H bonds that require overcoming an energy barrier of ∼100 kcal/mol.^3,8−11^ LPMOs are abundant in nature and categorized, based on their sequences, in seven distinct families (AA9-AA11 and AA13-AA16), within the class of auxiliary activities (AAs) in the CAZy database.^12^ Central to LPMO action is a unique mononuclear copper-active site made up of two histidines, where the N-terminal histidine coordinates with both the imidazole ring and the N-terminal amine.^8,13^ When reduced to Cu(I), LPMOs can activate O_2_^3,14^ or H_2_O_2_^11,15,16^ to create a reactive oxygen-containing intermediate that catalyzes the oxidation of glycosidic bonds in chitin,^3^ cellulose,^17^ and other plant-based polysaccharides^18−20^ (EC 1.14.99.53–1.14.99.56).

In the oxygen-driven mechanism, a fundamental challenge is the thermodynamically unfavorable formation of superoxide through reduction of O_2_ by Cu(I), a barrier that is potentially lowered by binding of the substrate.^14,21,22^ The formed superoxide may react as the oxidant or can be further reduced to create a Cu(II)-oxyl or Cu(III) hydroxide.^9,10,23,24^ Several catalytic scenarios have been proposed for the H_2_O_2_-driven peroxygenase reaction.^25^ Accumulating data from experiments and modeling indicate that the peroxygenase reaction entails homolytic cleavage of H_2_O_2_ by the reduced LPMO, leading to the formation of a hydroxyl radical that may react directly with the substrate or generate a Cu(II)-oxyl species.^11,15,25^

While there is some debate in the field as to the relative importance of the monooxygenase and peroxygenase reaction in nature, it is evident that the peroxygenase reaction is orders of magnitude faster.^26−29^ For example, the first kinetic characterization with respect to O_2_ showed an apparent oxidative rate of 0.02 s^–1^ for a bacterial chitin-active AA10.^3^ An in-depth kinetic analysis of the same LPMO revealed that the *k*_cat_ value for chitin oxidation increased to 6.7 s^–1^ when H_2_O_2_ was used as the co-substrate.^30^ Furthermore, with a Michaelis constant (*K*_m_) for H_2_O_2_ in the low μM range (2.8 μM), the enzyme has an efficiency constant (*k*_cat_/*K*_m_) of ∼10^6^ M^–1^ s^–1^, which is similar to the efficiency constants reported for heme-dependent peroxygenases.^30,31^

LPMOs are widespread in nature, in particular in fungi, some of which contain over 50 LPMO genes.^32^ While the role of some of these LPMOs in biomass conversion is well established, supported by both enzymological and expression data as well as successful use in industrial biomass conversion,^33^ the biological roles of many of these (putative) LPMOs remain enigmatic. It is noteworthy that the majority of characterized bacterial LPMOs are active on chitin, whereas several of these enzymes come from bacteria whose ecological niches do not suggest involvement in chitin degradation.^34^ This may be taken to suggest that chitin is not the true substrate of some of these enzymes. A considerable fraction of LPMOs contain one or more additional domains. Although in some cases these domains are known to be involved in chitin or cellulose binding, several are predicted to be involved in binding other materials or have unknown functions.^35^ The discovery of a starch-active LPMO cleaving α-glycosidic bonds^19^ gave one glimpse of a larger functional diversity that may exist among LPMOs. Functional diversity is also suggested by variation in the shapes of the substrate-binding surfaces that vary from being flat and having aromatic surface residues, matching well with binding crystalline polysaccharide substrates, to being more convex and/or polar^36−38^ (Figure S1).

In search for functional diversity, we turned our attention to putatively chitin-active AA11 LPMOs. The substrate-binding surface of the only structurally characterized member of this family, *Ao*AA11 from *Aspergillus oryzae*,^36^ is more convex compared to bacterial chitin-active LPMOs (AA10s) (Figure S1) and is free of aromatic residues, where the latter are known to be important for substrate binding in chitin-active AA10s.^39,40^ Secretome data for *Aspergillus fumigatus* show that at least three AA11s are expressed.^41^ The catalytic domains of one of these, *Af*AA11B, shares 72.6% sequence identity with *Ao*AA11 (Figure S1), whereas the other two, *Af*AA11A and *Af*AA11C, are less similar to *Ao*AA11, with 39.6% and 37.5% identity, respectively. Initial functional screening of several of these AA11 LPMOs revealed that *Af*AA11B had remarkable and hitherto never described activity on soluble chito-oligosaccharides. In-depth functional characterization of *Af*AA11B revealed multiple unusual LPMO features, such as a low redox potential, high oxidase activity, and strong preference for soluble substrates, suggesting involvement of this AA11 in processes other than chitin degradation. Furthermore, competition experiments with horseradish peroxidase (HRP) showed that the monooxygenase activity of this LPMO is essentially non-existent.

## Materials and Methods

### Cloning

Cloning of *Af*AA11B was done as described before.^42^ Briefly, the synthetic *Af*AA11B gene (NCBI accession number XP_748042.1) including its native signal sequence was codon optimized for *Pichia pastoris* (GenScript, NY, USA), excised from the pUC57 vector, and ligated into the pPINK-GAP vector,^43^ yielding in the pPINK-GAP_ *Af*AA11B plasmid.

pPINK-GAP_ *Af*AA11B was transformed to *P. pastoris* PichiaPink Strain 4 cells, following the manufacturer’s instructions (Invitrogen, CA, USA). Transformants were screened for protein production in buffered complex glycerol medium (containing 1% (v/v) glycerol), which was prepared according to the manufacturer’s instructions (Invitrogen, CA, USA). The best-producing transformant was used for the expression of recombinant *Af*AA11B used in the presented study.

### Expression and Purification

A single yeast colony was used to inoculate 25 mL of BMGY (1% (v/v) glycerol) in a 100 mL baffled shake flask, and the culture was incubated at 30 °C and 150 rpm for 24 h. This pre-culture (12.5 mL) was used to inoculate 500 mL of buffered minimal medium containing 1.34% YNB, 0.00004% biotin, 100 mM potassium phosphate (pH 6.0), 0.5% (w/v) glucose, and 0.5% (v/v) glycerol in a 2 L baffled shake flask. The culture was incubated at 30 °C and 150 rpm for 48 h. After 24 h, 0.25% (v/v) glycerol and 0.25% (w/v) glucose were added.

Cells and debris were removed by centrifugation at 10,000*g* for 15 min at 4 °C. The protein-containing supernatant was filtered with a 45 μm Steritop bottle-top filter (Merck Millipore, Burlington, MA, USA) and concentrated 5-fold by using a VivaFlow 200 tangential crossflow concentrator (molecular weight cut-off, MWCO 10 kDa, Sartorius Stedim Biotech Gmbh, Germany) prior to protein purification.

Ammonium sulfate was added to the concentrated culture supernatant to a final concentration of 2.4 M before loading onto a 5 mL HiTrap Phenyl FF column (GE Healthcare Life Sciences, Uppsala, Sweden), which was equilibrated with 50 mM of bis-tris/HCl buffer (pH 6.5), containing 2.4 M ammonium sulfate. The protein was eluted from the column by applying a 35 mL linear gradient from 2.4 to 0 M ammonium sulfate in 50 mM bis-tris/HCl buffer (pH 6.5) using a flow rate of 1.8 mL/min. The collected fractions were analyzed by sodium dodecyl sulfate polyacrylamide gel electrophoresis (SDS-PAGE) and fractions showing a protein band of the correct size were pooled. Prior to subsequent purification steps, the buffer was exchanged to 20 mM tris/HCl (pH 8.4) by using Amicon Ultra centrifugal filters (MWCO 10 kDa, Merck Millipore, Burlington, MA, USA).

The salt-free protein solution was loaded onto a 5 mL HiTrap DEAE FF column (GE Healthcare Life Sciences, Uppsala, Sweden) that was equilibrated with 20 mM tris/HCl (pH 8.4). The protein was eluted by applying a 100 mL linear gradient from 0 to 30% 0.5 M NaCl in 20 mM tris/HCl (pH 8.4) using a flow rate of 1.8 mL/min.

The collected fractions were analyzed by SDS-PAGE and pooled if the target protein was present, followed by concentrating to 1.5 mL using Amicon Ultra centrifugal filters (MWCO 10 kDa, Merck Millipore, Burlington, MA, USA). The concentrated protein solution was loaded onto a HiLoad 16/60 Superdex 75 size exclusion column (GE Healthcare Life Sciences, Uppsala, Sweden) in 50 mM bis-tris/HCl (pH 6.5), containing 150 mM NaCl, using a flow rate of 0.75 mL/min. Fractions containing the enzyme were identified using SDS-PAGE, pooled, and concentrated using Amicon Ultra centrifugal filters (MWCO 10 kDa, Merck Millipore, Burlington, MA, USA).

### Copper Saturation and Quantification

For copper saturation, the LPMO was incubated with a 3-fold molar excess of CuSO_4_ for 60 min on ice. Unbound copper was removed, and buffer was exchanged by washing with 50 mM bis-tris/HCl (pH 6.5) using Amicon Ultra centrifugal filters (MWCO 10 kDa, Merck Millipore, Burlington, MA, USA). The copper content in the resulting LPMO samples was assessed by ICP–MS. The purity of the protein was analyzed by SDS-PAGE (Figure S2). The *A*_280_ method was used to determine the protein concentration using the theoretical extinction coefficient (ε = 40450 M^–1^ cm^–1^). The purified and copper-saturated enzyme was stored at 4 °C.

### LPMO Reactions

For analysis of enzyme activity, 200 μL of reaction mixtures was prepared in 1.5 mL reaction tubes with conical bottom. Standard LPMO reactions contained 1 μM of LPMO, 2 mM of *N*-acetyl-chito-oligosaccharides (Megazyme; 95% purity), or 15 g/L of crystalline chitin (α-chitin from Chitinor Seagarden/Tromsø, Norway) and β-chitin from France Chitin (Orange, France). As a reductant, 1 mM of l-ascorbic acid (AscA; Sigma-Aldrich) was used. All reactions were carried out in 50 mM of bis-tris/HCl (pH 6.5) and incubated at 37 °C and 750 rpm in a Thermomixer C (Eppendorf, Hamburg, Germany). Standard reactions with hydrogen peroxide (37% (v/v) stock solution, Merck) contained 300 μM of H_2_O_2_. Stock solutions of AscA and H_2_O_2_ with concentrations of 50 and 10 mM, respectively, were prepared in pure water (TraceSELECT, Fluka) and stored at −20 °C. Prior to use, the concentration of the H_2_O_2_ stock solution was verified by measuring absorbance at 240 nm and using a molar extinction coefficient of 43.6 M^–1^ cm^–1^. The conditions used in non-standard activity assays are described in the Results section in the corresponding figure texts.

Anaerobic experiments were performed inside an anaerobic chamber (Whitley A95 Workstation, Don Whitley Scientific Limited, UK). To ensure oxygen-free reactions, each reactant solution was separately prepared in an airtight GC vial (1.5 mL) and degassed by successive placing vacuum over the solution, followed by addition of oxygen-free nitrogen using a Schlenk line. Subsequently, reactant solutions were incubated inside the anaerobic chamber for at least 30 min prior setting up the reactions.

For time course experiments with soluble substrates, reaction conditions and timings were such that the substrate concentration in the samples would not go below 80% of the starting concentration. For sampling product formation, 25 μL of aliquots was withdrawn from the reaction and mixed with three volumes of 200 mM NaOH to quench the reaction. Reactions with crystalline chitin were terminated by a 10 min boiling step prior to degradation of the remaining solid chitin with a mixture of recombinantly produced purified chitinolytic enzymes from *Serratia marcescens*(44−46) [final concentrations: 2.5 μM chitinase A, 2.5 μM chitinase C, and 2 μM chitobiase] for 24 h at 37 °C and 150 rpm. Prior to product analysis, the reaction volumes were adjusted with 200 mM NaOH to quench the reaction and achieve a 4:1 dilution. Product solutions were obtained by filtering using a 96-well 0.45 μm filter plate (Merck Millipore, Billerica, MA) that was operated with a vacuum manifold. All experiments shown were done in at least three independent replicates.

### Detection of Oxidized Products

High-performance anion exchange chromatography with pulsed amperometric detection (HPAEC-PAD) and matrix-assisted laser desorption ionization–time-of-flight mass spectrometry (MALDI-ToF MS) were used to analyze oxidized products. HPAEC-PAD was conducted using a Dionex ICS5000 system equipped with a CarboPac PA1 analytical column (2 × 250 mm) and a CarboPac PA1 guard column (2 × 50 mm). Product separation was achieved by applying a 29 min gradient as previously described for cello-oligosaccharides (Figure S3).^47^ Oxidized products were quantified by using in-house made standards as described elsewhere.^46^ Chromatograms were recorded and analyzed with Chromeleon and plot preparation was done in Microsoft Excel. MALDI-ToF MS was performed on an Ultraflex MALDI-ToF/ToF instrument (Bruker Daltonik GmbH, Bremen, Germany) equipped with a Nitrogen 337 nm laser, as described previously.^18^

### Determination of the Redox Potential

The cell potential of the LPMO-Cu^2+^/LPMO-Cu^+^ redox couple was determined from the reaction between reduced *N*,*N*,*N*′,*N*′-tetramethyl-1,4-phenylenediamine (TMP_red_) and LPMO-Cu^2+^, as described previously.^39,48^ The concentrations of *Af*AA11B and TMP were 31 and 500 μM, respectively.

### H_2_O_2_ Production Assay

The capability of *Af*AA11B, *Sm*AA10A, and free copper to generate H_2_O_2_ was assessed as described by Kittl et al.^49^ The total reaction volume of 100 μL contained 1 μM of LPMO or CuSO_4_, 100 μM of Amplex Red, and 0.55 μM of HRP in 50 mM bis-tris/HCl (pH 6.5). After 5 min pre-incubation at 37 °C, the reactions were started by the addition of AscA to final concentrations of 50, 250, or 1000 μM. The generation of resorufin was measured by monitoring absorbance at 595 nm every 10 s over 3000 s in a plate reader. Blank reactions did not contain LPMO or CuSO_4_, and the calibration curves included AscA to incorporate the influence of the reductant on resorufin formation. The data shown was obtained from three independent replicates.

## Results

### Heterologous Expression of *Af*AA11B

The gene encoding for *Af*AA11B (NCBI accession number XP_748042.1) consists of 1257 base pairs encoding a secretion signal, the catalytic domain, and a linker region with an attached X278 module of unknown function (Figure S1).

The *Af*AA11B enzyme was recombinantly expressed in *P. pastoris* (*Komagataella phaffii*). SDS-PAGE analysis of the purified protein, obtained after several chromatographic steps, indicated a mass of approximately 60 kDa (Figure S2). As the theoretical calculated mass of *Af*AA11B is 42.8 kDa, it seems that the recombinant protein carries *N*- and/or *O*-glycosylations. The NetNGlyc and NetOGlyc online tools (http://www.cbs.dtu.dk/services) showed three potential N-glycosylation sites at positions Asn116, Asn134, and Asn228 and seven potential O-glycosylation sites at positions Ser174, Ser175, Ser192, Ser230, Ser241, and Thr143 in the catalytic domain. Another 47 potential O-glycosylation sites were identified for the linker region and the X278 module.

To obtain a structural impression of *Af*AA11B, the online tool SWISSMODEL (https://swissmodel.expasy.org) was used to generate a homology model of the catalytic domain based on the crystal structure of *Ao*AA11 (PDB: 4MAH;^36^) with 72.6% sequence identity (Figure S1). Notwithstanding uncertainties related to the two incomplete loop regions in the template structure (Figure S1, pink regions), the homology model of *Af*AA11B shows the classical immunoglobulin like β-sheet core and the surface-exposed copper coordinating histidine brace formed by the N-terminal histidine (His1) and the second histidine at position 61 in the mature protein with a tyrosine (Tyr141) in the proximal axial position of the copper center (Figure S1).

### Screening for LPMO Activity

Initial screening for LPMO activity included incubation of *Af*AA11B with α-chitin, β-chitin, cell walls of different yeast strains grown in different conditions (obtained from in-house fermentation processes), mannan from *Saccharomyces cerevisiae*, β-glucans from barley, Na-alginate, and cellopentaose, in the presence of molecular oxygen and 1 mM ascorbic acid. Products were only observed for the reactions with α- and β-chitin. MALDI-TOF-MS spectra showed signals corresponding to oxidized chito-oligomers of varying lengths (DP3-DP7 with a mass difference of 203). The dominating signals corresponded to aldonic acids in the mono and double sodium adduct form (Figure S4), showing that *Af*AA11B, cleaves the glycosidic bonds by oxidizing the C1 position. It is noteworthy that the mass spectrum contains multiple additional signals that reflect unknown compounds as well as partially deacetylated oxidized chito-oligosaccharides. Most of these additional signals did not appear in MS analysis of products generated in a control reaction with the well-studied bacterial LPMO, *Sm*AA10A.^3^

**Figure 1:**
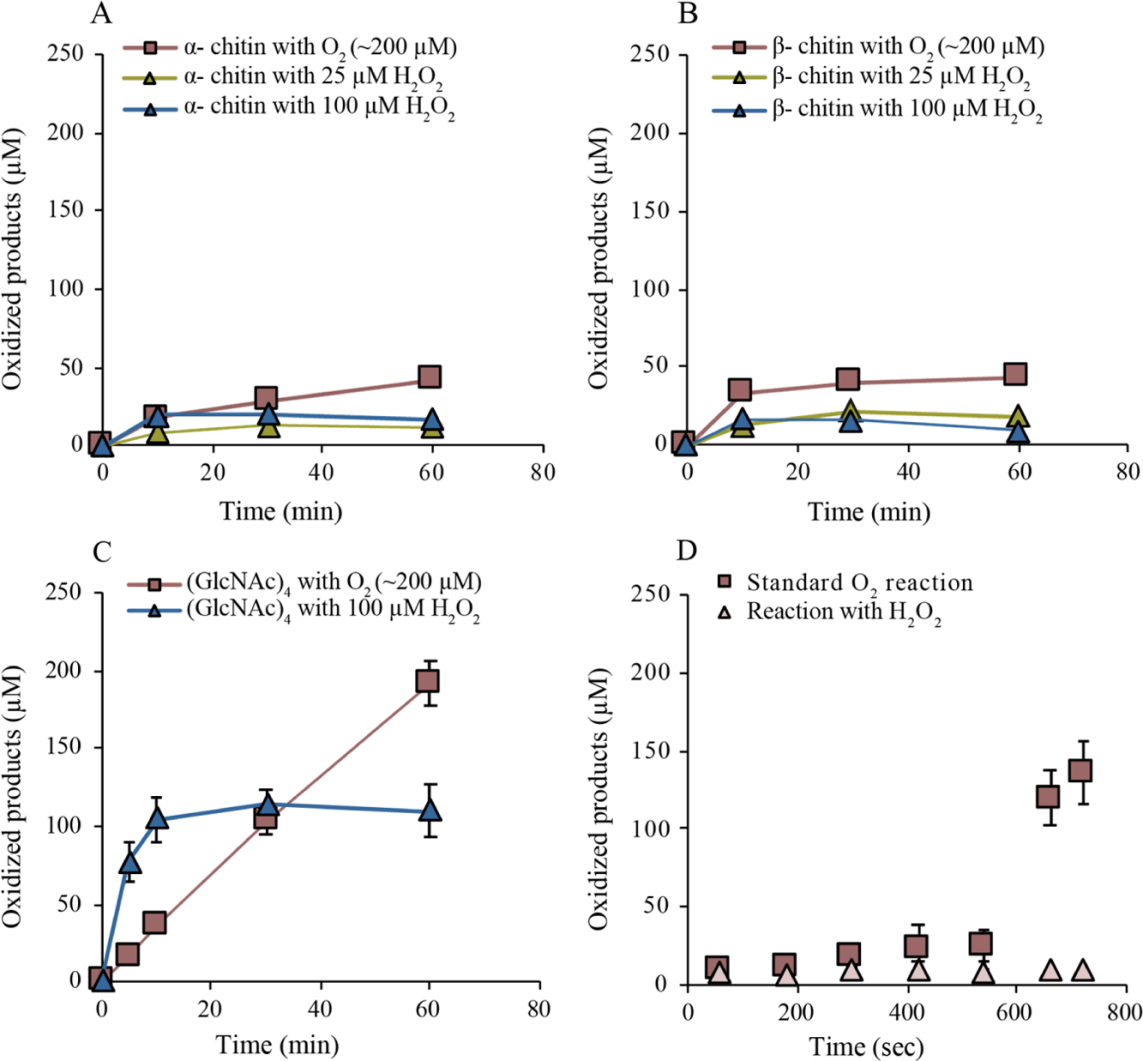
Time course experiments showing the formation of oxidized products by *Af*AA11B in different reactions (see Materials and Methods for details). In (A,B), α- and β-chitin (15 g/L) were used as substrates, respectively. Boxes show data from standard reactions, whereas triangles show data from reactions which were supplemented with different H_2_O_2_ concentrations in the presence of 20 μM AscA, as indicated in the figure. (C) Reactions with (GlcNAc)_4_; one standard reaction (boxes) and one reaction with 100 μM H_2_O_2_ and 20 μM AscA. (D) Standard aerobic reaction with β-chitin and 1 mM AscA (boxes) and an aerobic reaction with β-chitin in the presence of 300 μM H_2_O_2_ and 20 μM AscA (triangles). After 10 min (line), fresh H_2_O_2_, (GlcNAc)_4_, and AscA were added to final concentrations of 300 μM, 2 mM, and 1 mM, respectively. The data points represent the average values of at least two independent experiments; vertical lines, which sometimes are hidden by the data points, indicate standard deviations. Control experiments in which samples were pre-incubated in the absence of chitin showed no product formation up to 10 min; after the addition of fresh (co-)substrates, no product formation was observed for the reaction pre-incubated with H_2_O_2_, whereas the reaction pre-incubated under standard aerobic conditions showed strongly reduced product formation (about 17 μM after 60 s) relative to the reaction pre-incubated with chitin.

Time course analyses of the degradation of α- and β-chitin under conditions typically used for LPMO characterization, that is, in the presence of O_2_ and 1 mM ascorbic acid, showed non-linear product formation curves and yielded approximately 50 μM of oxidized products after 60 min incubation, for both substrates (Figure 1A,B). Reactions with the addition of 20 or 100 μM H_2_O_2_ and containing only priming amounts of AscA (20 μM) showed early cessation of product formation with ∼15 μM product being formed within the first 10 min of the experiment (Figure 1A,B). Of note, the chitin concentration used in these experiments corresponds to a tetramer concentration of approximately 18 mM, indicating that only a tiny fraction of the chitin was oxidized. Under similar standard conditions (O_2_, 1 mM AscA), chitin-active AA10 LPMOs may produce on the order of 1 mM of oxidized products.^50^

In stark contrast to the results above, a standard reaction of *Af*AA11B with 2 mM soluble (GlcNAc)_4_ yielded a linear progress curve, reaching ∼200 μM of oxidized product after 60 min (Figure 1C). The use of H_2_O_2_ (100 μM) in the presence of a priming amount of AscA (20 μM) led to an increased rate of oxidation and formation of ∼100 μM oxidized product within 10 min (Figure 1C). These observations suggest that soluble (GlcNAc)_4_ is a better substrate than solid chitin for *Af*AA11B and that, in reactions with the more preferred substrate, (GlcNAc)_4_, H_2_O_2_ is a better co-substrate than O_2_. The data for the reaction with H_2_O_2_ suggest that H_2_O_2_ was stoichiometrically converted to oxidized product.

The inability of *Af*AA11B to catalyze oxidation of α- and β-chitin in the presence of O_2_ or H_2_O_2_ can either be due to enzyme inactivation or to limitations in substrate access. To assess this, we set up a standard reaction with β-chitin (aerobic, 1 mM AscA) as well as an aerobic reaction with 300 μM H_2_O_2_ and 20 μM AscA. After 10 min of incubation, (GlcNAc)_4_, to 2 mM, H_2_O_2_, to 300 μM, and AscA, to 1 mM, were added to both reaction mixtures to verify whether the LPMO was still catalytically competent. In the reaction with H_2_O_2_ as the initial co-substrate, no newly formed oxidized products were observed after adding (GlcNAc)_4_ (Figure 1D), suggesting that *Af*AA11B had been deactivated under these conditions because of non-productive reactions with H_2_O_2_. Apparently, whereas this LPMO can productively use large amounts of added H_2_O_2_ to degrade soluble substrates (Figure 1C; more data below), it cannot do so when the substrate is chitin. In the standard reaction with O_2_ as the co-substrate, the formation of oxidized products drastically accelerated after adding the soluble substrate (Figure 1D), showing that under these standard conditions, the enzyme remains active. This again suggests that the limited product yields in standard aerobic reactions with insoluble chitin are due to the limited access to the substrate and not to enzyme inactivation. A control standard aerobic reaction where chitin was left out during the pre-incubation led to considerable enzyme inactivation (Figure 1D). This is to be expected since pre-incubation without chitin will lead to generation of H_2_O_2_^49^ that will react non-productively with the LPMO, leading to enzyme inactivation.

To further asses the ability of *Af*AA11B to catalyze the oxidation of soluble substrates, the enzyme was incubated with chitin oligomers (2 mM) with different degrees of polymerization (DP) ranging from 3 to 6 in the presence of H_2_O_2_ (100 μM) and AscA (20 μM). The rate of reaction was determined from linear progress curves for the formation of oxidized products over time. The enzyme was active on all tested substrates, and the highest observed rate constant (*k*_obs_) was measured for (GlcNAc)_4_ with a value of 0.245 ± 0.007 s^–1^ (Table 1).

**Table 1:** Observed Oxidation Rates and Binding Modes for *Af*AA11B Acting on Chitin Oligomers (2 mM) with Different DP in the Presence of 100 μM H_2_O_2_ and 20 μM AscA^a^

	*k*_obs_ (s^–1^)	mode of binding			
DP3	0.145 ± 0.005	–2 → +1			
		100%			
DP4	0.245 ± 0.007	–2 → +2	–3 → +1		
		82%	18%		
DP5	0.169 ± 0.013	–2 → +3	–3 → +2	–4 → +1	
		58%	28%	14%	
DP6	0.154 ± 0.006	–2 → +4	–3 → +3	–4 → +2	–5 → +1
		47%	23%	24%	6%

a Rates were determined by measuring the generation of oxidized products over time. Note that the kinetic analysis described further below show that the rates reported in this table are far below the maximum rates due to a sub-saturating reductant concentration. The binding modes were determined by calculation of the relative rates of appearance of oxidized products of the different lengths. The numbers in the “Mode of binding” columns refer to subsites–subsites interact with the non-reducing end of the substrate.

To obtain insights into preferred substrate binding modes, we studied product profiles obtained in reactions of *Af*AA11B with 2 mM of chitin oligomers with varying DP (DP2-DP6) in the presence of H_2_O_2_ (100 μM) and AscA (20 μM). After a 30 s turnover, the reaction was quenched and analyzed by HPAEC-PAD, and the relative abundance of the different oxidized products was calculated based on the recorded chromatograms. The results showed that the oxidized dimer is the dominant oxidized product, regardless of the length of the oligomeric substrate (Table 1). This suggests that all substrates bind strongly to subsites −2 and −1 [following the subsite nomenclature previously used to describe the interaction of glycoside hydrolases^51^ and LPMOs^21,26^ with their substrates] and that binding to these subsites is essential for productive substrate binding (Table 1). Based on the relative appearance of each oxidized product, it was possible to establish a rudimentary overview of preferred binding modes (Table 1). Of note, multiple cleavages of the longer substrates cannot be excluded, and the preferred binding modes given in Table 1 may thus differ from reality. However, the product peaks together corresponded to as little as ∼10–15 μM of oxidized product (at an initial substrate concentration of 2 mM and H_2_O_2_ concentration of 100 μM), which shows that the initial rate conditions were met.

### Detailed Kinetic Analysis of *Af*AA11B-Catalyzed Oxidation of (GlcNAc)_4_

The interesting observations that *Af*AA11B prefers soluble chitinous substrates and works more efficiently in the presence of added H_2_O_2_ prompted us to undertake a detailed kinetic analysis of (GlcNAc)_4_ oxidation. *Af*AA11B turnover under standard conditions, that is, in the presence of atmospheric O_2_, (GlcNAc)_4_ (2 mM), and AscA (1 mM) yielded an observed rate constant (*k*_obs_) of 0.052 ± 0.004 s^–1^ (calculated from data shown in Figure 1C), which is about 5 times lower than the *k*_obs_ value for the reaction with 100 μM H_2_O_2_ and 20 μM AscA (Table 1).

It is well known that H_2_O_2_ accumulates in reactions that contain an LPMO and a reductant but no LPMO substrate.^49,52^ It has been suggested that this H_2_O_2_-generating oxidase activity also plays a role in reactions with the substrate, where LPMOs could generate their own co-substrate.^25^ Although it seems certain that reduced LPMOs react with oxygen,^11,14,22^ there is debate in the field regarding the occurrence and kinetic relevance of a true monooxygenase reaction; that is, a reaction where the substrate-oxidizing reactive oxygen species is generated directly from O_2_, in the active site of the substrate-bound LPMO. To gain more insights into these issues, we first assessed the H_2_O_2_-generating ability of *Af*AA11B.

**Figure 2:**
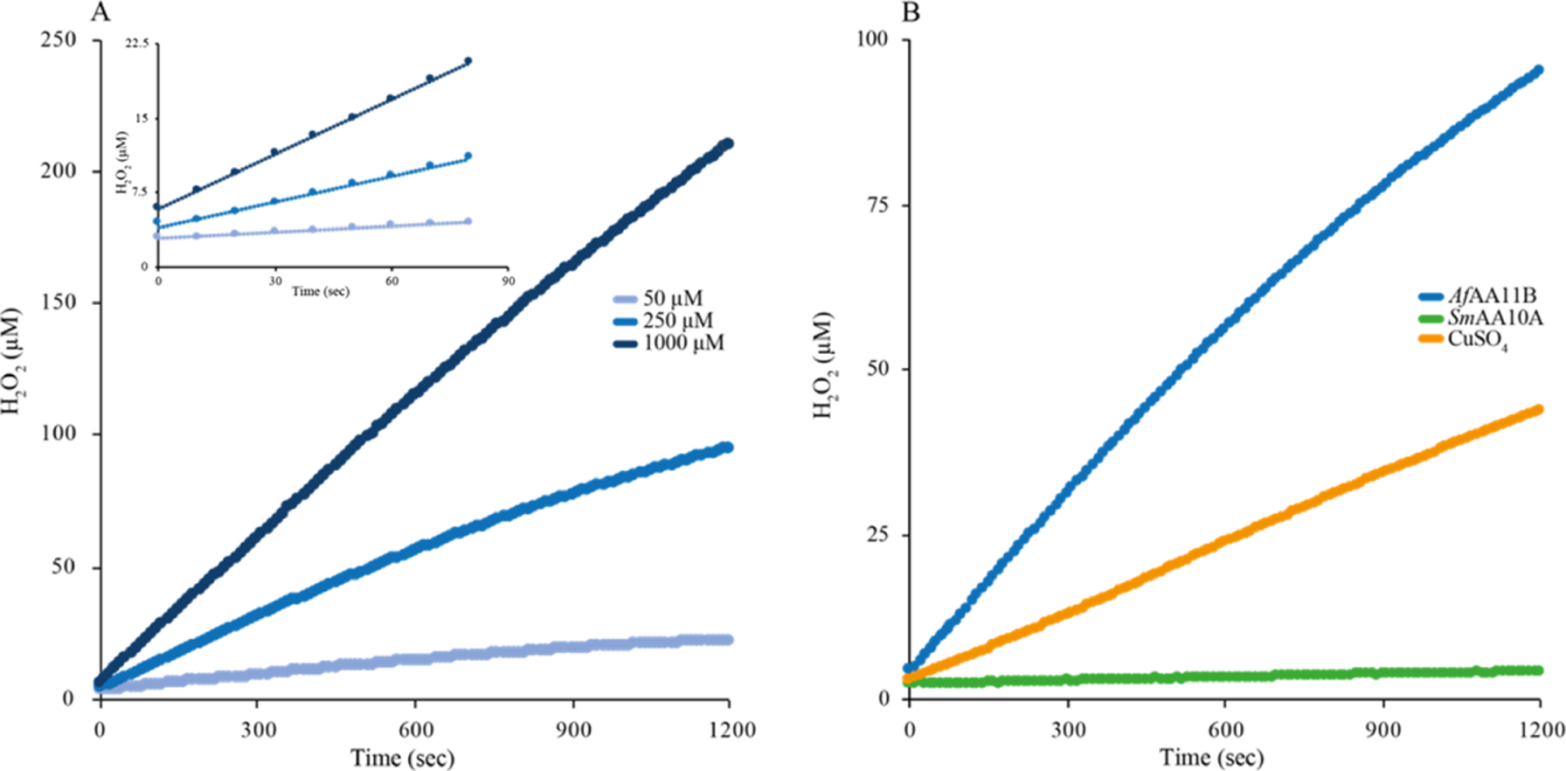
H_2_O_2_ production curves. (A) H_2_O_2_ production by 1 μM *Af*AA11B in the presence of 50, 250, or 1000 μM AscA. The inset shows data for the first 80 s of the reaction. (B) Comparison of H_2_O_2_ production by 1 μM of *Af*AA11B, *Sm*AA10A, or CuSO_4_ in the presence of 250 μM reductant. H_2_O_2_ levels were calculated after correcting for side reactions involving AscA and Amplex Red by using a H_2_O_2_ standard curve that was prepared in the presence of the same amount of reductant (no LPMO/CuSO_4_).

In the presence of 50 μM AscA, the observed initial rate of H_2_O_2_ production by 1 μM *Af*AA11B was 0.017 ± 0.001 μM*s^–1^, which is higher than the H_2_O_2_ production rate for 1 μM free Cu(II) under the same conditions (0.008 ± 0.001 μM*s^–1^; Figure 2 and Table 2). Upon increasing the AscA concentration to 1000 μM, the rates increased to 0.183 ± 0.016 and 0.080 ± 0.002 μM*s^–1^ for *Af*AA11B and free Cu(II), respectively. It is noteworthy that the rate of the standard LPMO reaction (0.052 ± 0.004 μM*s^–1^; Figure 1C) is lower than the rate of H_2_O_2_ production (0.183 ± 0.016 μM*s^–1^; Table 2). It is plausible that LPMO generates less H_2_O_2_ because the oxidase reaction is inhibited by interactions with the substrate or that the produced H_2_O_2_ is at such low concentration that *V*_max_ is not achieved.^49,52^

**Table 2:** Observed Rate Constants for Production of H_2_O_2_ by *Af*AA11B, *Sm*AA10A, and CuSO_4_, all at 1 μM Concentration, in the Presence of Different Reductant Concentrations^a^

	observed rate (μM*s^–1^)		
[AscA] μM	*Af*AA11B (*E*_0_ = 0.114 V)	CuSO_4_ (*E*_0_ = 0.160 V)	*Sm*AA10A (*E*_0_ = 0.275 V)
50	0.017 ± 0.001	0.008 ± 0.001	0.001 ± 0.001
250	0.091 ± 0.006	0.034 ± 0.002	0.002 ± 0.001
1000	0.183 ± 0.016	0.080 ± 0.002	0.001 ± 0.001

a The redox potentials for the Cu(II)/Cu(I) redox couples are indicated in the column headers. The signals obtained in the Amplex Red signal were corrected for the effect of ascorbic acid^28^ and the rates were corrected for the rate in reactions with only ascorbic acid.

Since the first step in H_2_O_2_ production, formation of O_2_^•–^, is endergonic, it is interesting to compare the redox potentials to see if there is a correlation between these potentials and the ability to produce H_2_O_2_. In accordance with the high apparent oxidase activity, the redox potential of the *Af*AA11B-Cu(II)/*Af*AA11B-Cu(I) redox couple, determined as described previously,^39,48^ was found to be of 114 ± 1 mV, that is, lower than the literature value for the Cu(II)/Cu(I) redox couple of 160 mV. In comparison, chitin-active *Sm*AA10A has a redox potential for the *Sm*AA10A-Cu(II)/*Sm*AA10A-Cu(I) redox couple of 275 mV^39^ and reactions containing 1 μM *Sm*AA10A showed very low H_2_O_2_ production rates of 0.001 ± 0.001 μM*s^–1^, at both the tested AscA concentrations of 50 and 1000 μM, respectively (Figure 2 and Table 2).

**Figure 3:**
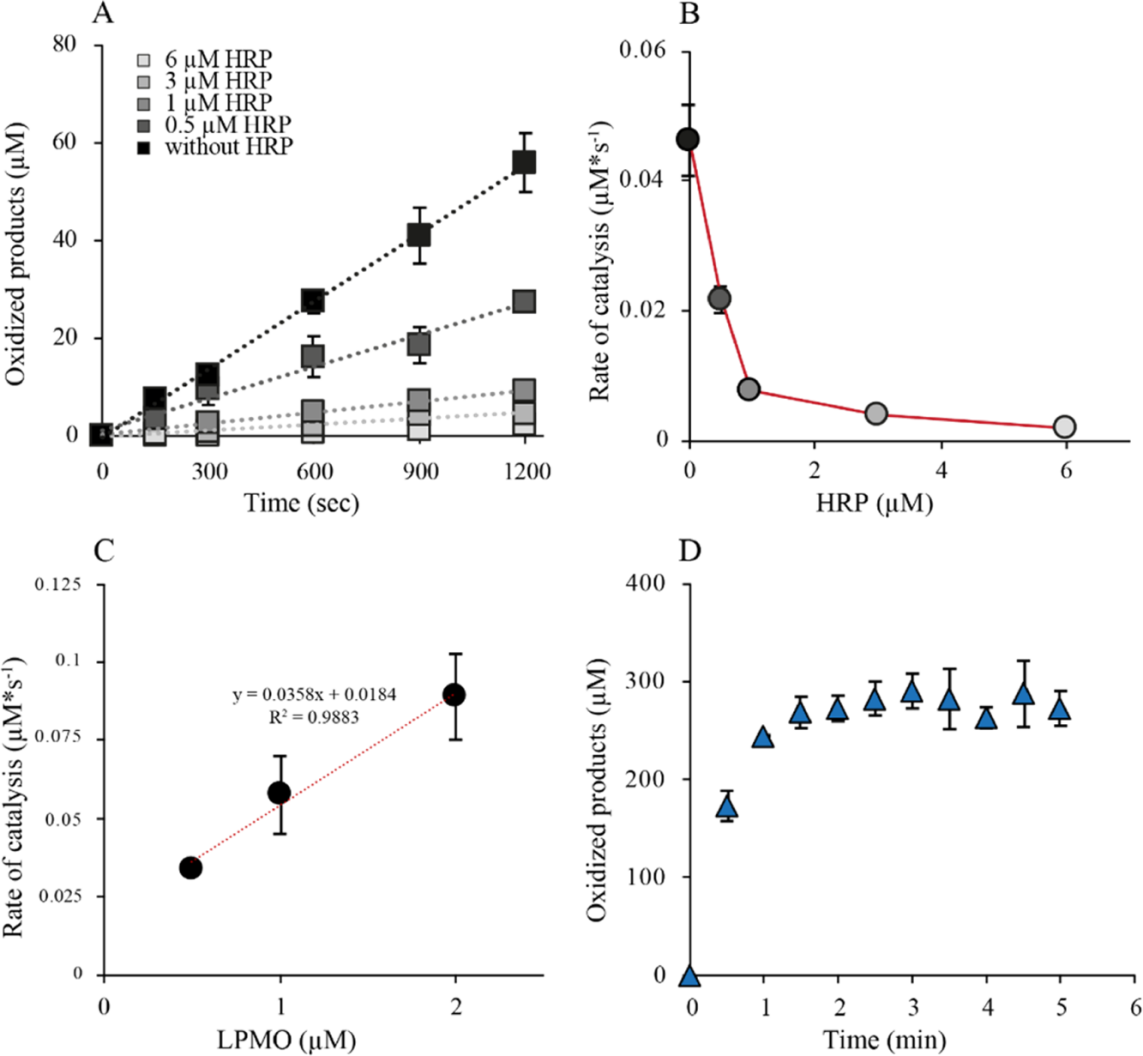
Inhibition of substrate oxidation by HRP. (A) Progress curves for reactions with 1 μM LPMO, 1 mM AscA, 2 mM (GlcNAc)_4_, 100 μM Amplex Red, and different concentrations of HRP. (B) Plot of the reaction rates obtained from (A) against the HRP concentration, showing that the reaction rate approaches zero at high HRP concentrations. (C) Observed rates of standard reactions as in (A) using different LPMO concentrations. (D) Anaerobic time course experiment with 1 μM LPMO in the presence of 300 μM H_2_O_2_, (GlcNAc)_4_ (2 mM), and AscA (1 mM).

The connection between the generation of H_2_O_2_ and substrate oxidation by *Af*AA11B was investigated by studying the ability of HRP to inhibit substrate oxidation. Standard oxygen reactions with high concentrations of soluble substrate (2 mM) and varying concentrations of HRP resulted in linear progress curves, showing that the rate of substrate oxidation decreased with increasing HRP concentration (Figure 3A). Importantly, the linearity of the progress curves shows that depletion of AscA by HRP is not responsible for the inhibition of *Af*AA11B under these conditions. Plotting of the reaction rates against the HRP concentration gave a reversed hyperbolic curve showing 50% inhibition of LPMO activity at an LPMO/HRP ratio of 1:0.5 and 95% inhibition at a 1:6 ratio (Figure 3B).

Determination of the rate of (GlcNAc)_4_ oxidation in the presence of O_2_ at atmospheric pressure and AscA (1 mM) at varying LPMO concentrations showed a linear correlation between the enzyme concentration and product yields (Figure 3C). This shows that indeed LPMO is limiting the apparent monooxygenase reaction, which is in contrast to observations made for other LPMOs whose reactions may be limited by H_2_O_2_-generating LPMO-independent side reactions involving the reductant and O_2_.^53^ Importantly, the rate at the intercept (0 μM LPMO) was significant (0.0184 μM*s^–1^). Of note, this “LPMO-independent” background level of substrate conversion is effectively inhibited by HRP (Figure 3A,B), which shows that this conversion involves H_2_O_2_ generated in solution, likely resulting from auto-oxidation of ascorbic acid^53^ and is not due to, for example, a true monooxygenase reaction that would not involve H_2_O_2_.

To confirm that H_2_O_2_ generation, and not the peroxygenase reaction, limits the *Af*AA11B reaction, an anaerobic reaction was set up with (GlcNAc)_4_ (2 mM) AscA (1 mM) and a large amount of H_2_O_2_ (300 μM). This setup led to the rapid formation of 300 μM oxidized products within 1.5 min, demonstrating that the peroxygenase activity is much higher than the AscA oxidase activity (Table 2 and Figure 3D). This confirms that the reactions shown in Figure 3A–C indeed were H_2_O_2_-limited and further shows that *Af*AA11B, on average, is stable for a minimum of 300 peroxygenase turnovers.

**Figure 4:**
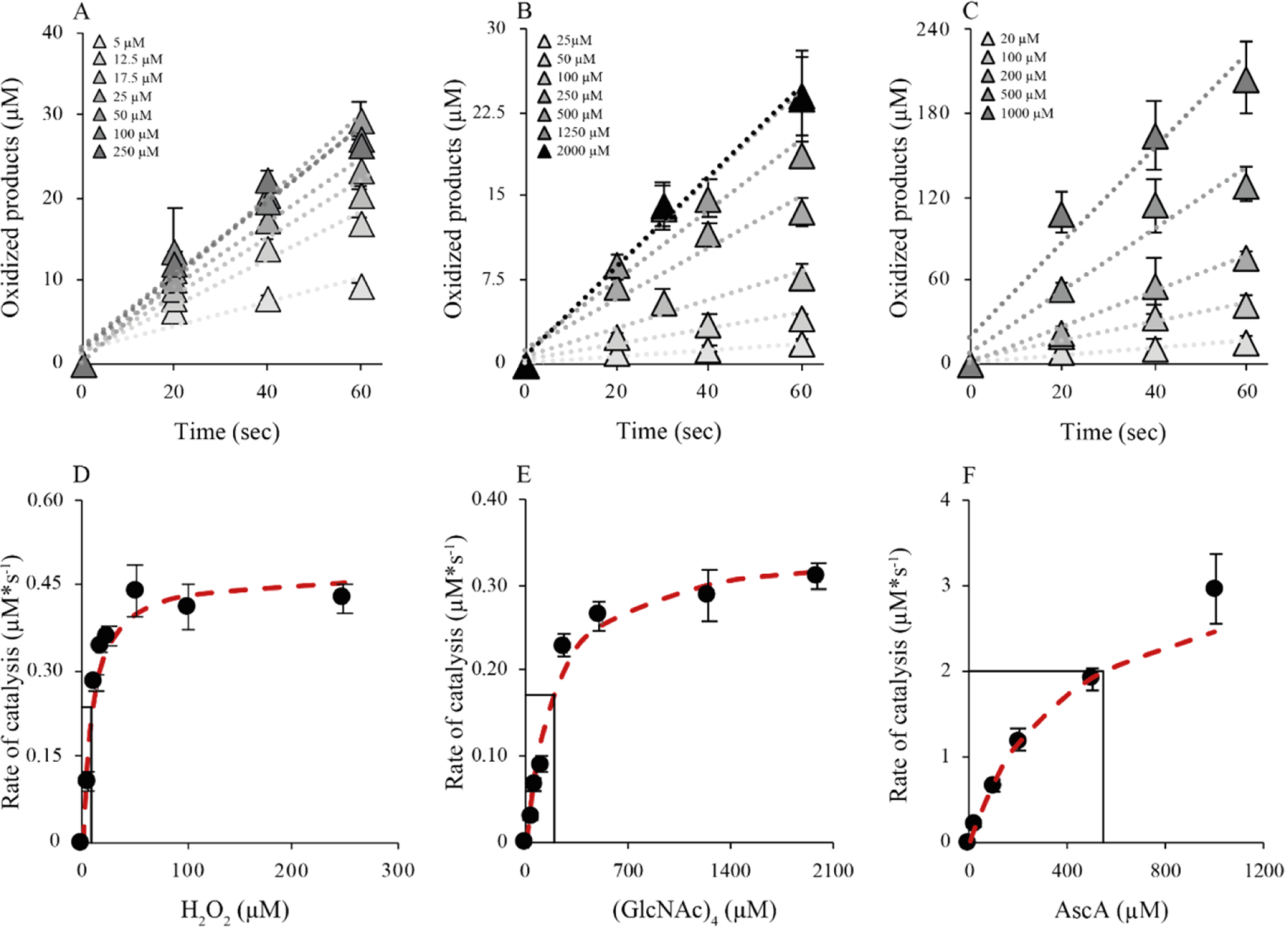
Michaelis–Menten kinetic analysis of *Af*AA11B. Panels (A–C) show progress curves, whereas panels (D–F) show the determined rates (black dots) as a function of the varied reaction parameter, with the fit to the Michaelis–Menten equation (red dashed line). All experiments were done in aerobic conditions. Conditions: panel (A/D), 0.1 μM LPMO, 1 mM AscA, 2 mM (GlcNAc)_4_, and varying H_2_O_2_ concentrations, as indicated; panel (B/E), 0.1 μM LPMO, 1 mM AscA, 300 μM H_2_O_2_, and varying (GlcNAc)_4_ concentrations, as indicated; and panel (C/F), 1 μM LPMO, 1 mM H_2_O_2_, 2 mM (GlcNAc)_4_, and varying AscA concentrations, as indicated.

To assess the peroxygenase activity of *Af*AA11B in detail, we determined the dependency of the initial enzyme rate on the concentration of (GlcNAc)_4_, H_2_O_2_, and AscA, and the data were analyzed using the Michaelis–Menten equation (Figure 4 and Table 3). All experiments were performed in aerobic conditions as the data above show that, under the used reaction conditions, the in situ generation of H_2_O_2_ from O_2_ (≤0.183 ± 0.016 μM*s^–1^, likely on the order of 0.052 ± 0.004 μM*s^–1^) in the presence of 2 mM (GlcNAc)_4_ is much lower than the rate of the peroxygenase reaction (on the order of 4 μM*s^–1^, Figure 3D).

**Table 3:** Kinetic Parameters of *Af*AA11B

*k*_cat_^a^	*K*_m_^H_2_O_2_^^b^	*k*_cat_/*K*_m_^H_2_O_2_^^c^	*K*_m_^(GlcNAc)_4_^^b^	*k*_cat_/*K*_m_^(GlcNAc)_4_^^c^	*K*_mR_^app^^b^
4.0 ± 0.6	8.9 ± 1.0	4.5 × 10^5^	200 ± 29	2.0 × 10^4^	502 ± 35

a s^–1^ (average value of three values; see text).

b μM.

c M^–1^ s^–1^.

Varying the H_2_O_2_ concentration in the presence of 2 mM (GlcNAc)_4_ and 1 mM AscA yielded a *k*_cat_ value of 4.7 ± 0.4 s^–1^ and a *K*_m_^H_2_O_2_^ value of 8.9 ± 1.0 μM. The assays for studying the dependency on the concentration of (GlcNAc)_4_ were performed in the presence of 300 μM H_2_O_2_ and 1 mM AscA and yielded a *k*_cat_ value of 3.5 ± 0.1 s^–1^ and *K*_m_^(GlcNAc)_4_^ of 200 ± 29 μM. Finally, the dependency of the initial enzyme rate on reductant concentration was determined using reactions with 1 mM H_2_O_2_ and 2 mM (GlcNAc)_4_ and yielded a *k*_cat_ value of 3.9 ± 0.2 s^–1^ and a *K*_m_ value of 502 ± 35 μM. The *k*_cat_ value reported in Table 3 (4.0 ± 0.6 s^–1^) is the average of the three values reported above. Of note, the *K*_m_ value for AscA should be viewed as an apparent half-saturating concentration (*K*_mR_^app^)^54^ and will depend on the H_2_O_2_ concentration because the two compounds will react in what is a side reaction.

Having access to *k*_cat_ and *K*_m_ values, the efficiency constants (*k*_cat_/*K*_m_) for H_2_O_2_ and (GlcNAc)_4_ were calculated, yielding a *k*_cat_/*K*_m_^H_2_O_2_^ value of 4.5 × 10^5^ M^–1^ s^–1^ and a *k*_cat_/*K*_m_^(GlcNAc)_4_^ value of 2.0 × 10^4^ M^–1^ s^–1^, respectively.

## Discussion

Due to their importance in modern biorefineries and capability of catalyzing powerful redox chemistry, there is a vast interest in discovering and characterizing new LPMO activities. *A. fumigatus* expresses at least three AA11s where *Af*AA11B has low sequence identity with the other two, suggesting different biological roles. Previous work on *Ao*AA11, with a similar domain structure and 72.6% sequence identity in the catalytic domain, suggested that this enzyme is involved in chitin degradation,^36^ but functional characterization of *Ao*AA11 was limited in this previous study. The present data clearly show that chitin is not a bona fide substrate of *Af*AA11B. Product release from chitin by *Af*AA11B was minimal compared to well-known bacterial chitin-active LPMOs such as *Sm*AA10A.^3,50^ Importantly, the enzyme became rapidly deactivated by H_2_O_2_ in reactions with chitin (Figure 1D) but not in reactions with (GlcNAc)_4_ (Figure 3D). This supports the notion of chitin not being a true substrate since it is well known that binding to the substrate protects LPMOs from oxidative damage.^25,30,55^

At the same time, the ability of *Af*AA11B to stably turn over (GlcNAc)_4_ in the presence of large initial amounts of H_2_O_2_, which would result in inactivation of LPMOs on crystalline substrates,^25^ suggests that this oligomer is a good substrate. This is further supported by the kinetic analyses of the peroxygenase reaction with (GlcNAc)_4_, which yielded kinetic parameters that are in the same order of magnitude as those found for chitin-active LPMOs,^30^ cellulose-active LPMOs,^28^ and various hemeperoxygenases.^31^

We found that *Af*AA11B has additional functional features that make it stand out from other LPMOs. *Af*AA11B has the lowest redox potential observed for an LPMO so far (0.11 V). Existing data indicate that cellulose-active AA9s have redox potentials in the range from 0.19 to 0.22 V, cellulose-active AA10s have a redox potential near 0.25 V, and chitin-active AA10s have a redox potential around 0.28 V.^39,56−58^ Interestingly, *Af*AA11B has also an exceptional high oxidase rate. Although AA9 LPMOs^59^ and, even more so, AA10 LPMOs (Figure 2,^53^) produce less H_2_O_2_ compared to free copper in reactions with ascorbic acid, H_2_O_2_ production in the reaction with *Af*AA11B clearly surpassed H_2_O_2_ production in the reaction with free Cu(II). From the difference in redox potential, one can deduce that the thermodynamically unfavorable and likely rate-limiting reduction of O_2_ to superoxide will be accompanied by a 7.8 kcal/mol lesser energetic penalty in a reaction with *Af*AA11B compared to *Sm*AA10A. It will be interesting to see if the apparent correlation between a low redox potential and high oxidase activity that emerges from comparing *Af*AA11B and *Sm*AA10A is valid for all LPMOs.

The observed rate constant for an oxidase activity of 0.18 s^–1^ for *Af*AA11B (atmospheric O_2_ pressure and 1 mM AscA) is higher than the observed rate constant for (GlcNAc)_4_ oxidation (*k*_obs_ = 0.052 s^–1^) in the presence of the same amount of O_2_ and AscA. This may be taken to suggest that the oxidase activity of *Af*AA11B can support the apparent monooxygenase reaction in what, de facto, is a peroxygenase reaction. However, direct comparison of these rates is not valid because the oxidase activity of *Af*AA11B will likely be inhibited by the presence of the (GlcNAc)_4_ substrate. A further insight in this matter was obtained from the HRP inhibition experiments. Most importantly, with this LPMO, it was possible to show that HRP completely inhibits the LPMO activity, in conditions that are typically considered “monooxygenase” conditions (1 mM AscA, atmospheric O_2_). Thus, the apparent monooxygenase reaction is fueled only by H_2_O_2_ generated in solution (i.e., accessible to HRP) and not by O_2_ directly or by H_2_O_2_ that is formed in the enzyme–substrate complex but never leaves the active site (as has been suggested for an AA9 LPMO based on modeling studies^23^). We would thus argue that the monooxygenase reaction does not occur for this catalytically perfectly competent LPMO.

An interesting observation is the relatively high amount of AscA needed to keep *Af*AA11B half-saturated in the Cu(I) state during (GlcNAc)_4_ oxidation (*K*_mR_^app^ = 502 μM) resulting in an efficiency constant *k*_cat_/*K*_mR_^app^ of 8.0 × 10^3^ M^–1^ s^–1^. In comparison, the same values were 2 μM and 1.6 × 10^6^ M^–1^ s^–1^, respectively, for the peroxygenation reaction of *Sm*AA10A with insoluble chitin.^54^ This high value of *K*_mR_^app^ aligns well with the low redox potential of *Af*AA11B. On the one hand, the low redox potential will reduce the propensity of the reduction of the active site copper by AscA, while, on the other hand, it would promote oxidation of reduced LPMOs by O_2_ (oxidase activity) or H_2_O_2_ (peroxidase activity) in solution. The propensity of LPMOs to become re-oxidized in between subsequent peroxygenase reactions, and the resulting increased need for reductants, likely depends on substrate affinity. The observation that the enzyme shows linear progress curves (Figure 4) under conditions that, as suggested by the high *K*_mR_^app^, lead to considerable futile LPMO reoxidation seems contradictive to the notion that insufficient binding to the substrate results in enzyme inactivation (Figure 1D). There are, however, multiple possible explanations for this apparent contradiction. It is well known that not every interaction between reduced LPMO and H_2_O_2_ results in irreversible inactivation and that inactivation is slower than productive reactions^30^ In this respect, it is worth noting that the time scales of the inactivation experiment in Figure 1 and the progress curves of Figure 4 are quite different. Furthermore, it is plausible that some degree of LPMO inactivation did occur but remained undetected because the peroxygenase reaction is efficient and limited by the availability of H_2_O_2_.

The results described above demonstrate that a fungal LPMO in the AA family 11, which deviates in active site architecture from chitin-active bacterial LPMOs in the AA family 10, shows a high peroxygenase activity toward oligomeric GlcNAc in soluble form but is not capable of catalyzing oxidation of insoluble chitin. The unique functional properties of this LPMO and the notion that nature has other tools for cleaving chito-oligomers (chitinases and chitobiases) make one wonder about the true function of *Af*AA11B. Transcriptome data for *Neurospora crassa* showed the upregulation of an AA11 with an X278 module in the final stage of spore formation in the fruiting body,^60^ perhaps suggesting a role in cellular development. In preliminary experiments, we tested *Af*AA11B on a range of substrates, including chitin-containing cell walls, but were not able, potentially due to analytical limitations, to detect oxidized products. Further studies into this direction are warranted.

It is also worth considering whether the high oxidase activity of *Af*AA11B, facilitated by its low redox potential, could serve a biological purpose of its own. It is not easy for organisms to harness the chemical potential of copper because copper is rare, may easily precipitate (especially in its reduced form), and can engage in potentially damaging redox reactions (e.g., Fenton chemistry) if not properly controlled. Indeed, it has been proposed that LPMOs provide organisms with the opportunity to harness and control the power of Fenton chemistry in biomass degradation.^25^ It might be that *Af*AA11B provides the organism with a tool to produce H_2_O_2_ in a process that would be controlled by the delivery of reducing equivalents.

In conclusion, the present results clearly show fast peroxygenase reactions catalyzed by *Af*AA11B, suggesting that this enzyme is indeed a true peroxygenase and not a monooxygenase. The fact that the presence of the oligomeric substrate is required for fast and stable LPMO reactions to occur in the presence of high concentrations of H_2_O_2_ suggests that these soluble substrates are bona fide LPMO substrates. On the other hand, the notion that nature may achieve cleavage of chito-oligomers using common hydrolytic enzymes leaves one wondering about the true biological role of *Af*AA11B, as alluded to the above.

The present findings support previous claims made by some that the apparent monooxygenase activity of LPMOs in general not only is exceedingly slow^15,27^ but possibly non-existent.^10^ The general picture, emerging from studies on multiple bacterial and fungal LPMOs, is that these enzymes are effective peroxygenases.^16,28,30^ It remains, however, difficult to fully exclude a monooxygenase reaction because it is difficult to create “monooxygenase conditions” that do not lead to in situ generation of H_2_O_2_ and because LPMOs may have varying catalytic properties. As to the latter, next to demonstrating a novel LPMO functionality, efficient cleavage of soluble chito-oligomers, our data show that, despite the conserved copper histidine brace, LPMOs show considerable variation in redox potential. Unraveling the molecular basis and biological implications of these differences in redox potential may provide important novel insights into copper biochemistry.

## Supporting Information Available

Materials and methods, results, and discussion for the Eyring analysis of the peroxygenase activity of *Af*AA11B, overview of sequence and structure of *Af*AA11B and comparison with other LPMOs, SDS-PAGE gel of purified *Af*AA11B, typical HPAEC-PAD chromatograms of product formation catalyzed by *Af*AA11B, and MALDI-ToF-MS spectra of product formation catalyzed by *Af*AA11B (PDF)
