# Supplementary material for: Kinetic Characterization of a Putatively Chitin-Active LPMO Reveals a Preference for Soluble Substrates and Absence of Monooxygenase Activity
Source: ACS Catal. 2021 Sep 7;11(18):11685–95. doi: 10.1021/acscatal.1c03344 (PMC8453653; doi:10.1021/acscatal.1c03344)
Supplement: Supplementary file 1 — cs1c03344_si_001.pdf [file cs1c03344_si_001.pdf]

Supporting Information for

Characterization of a Putatively Chitin-Active LPMO Reveals a Preference for Soluble Substrates and Absence of Monooxygenase Activity

Lukas Rieder[1], Dejan Petrović[1], Priit Väljamäe[2], Vincent G.H. Eijsink[1], Morten Sørlie[1*]

[1]Faculty of Chemistry, Biotechnology, and Food Sciences, Norwegian University of Life Sciences (NMBU), N-1432 Ås, Norway

[2] Institute of Molecular and Cell Biology, University of Tartu, 50090 Tartu, Estonia

*Corresponding author: Morten Sørlie

Email: morten.sorlie@nmbu.no

Contents:

Supporting Materials and Methods (Eyring analysis)

Figures S1 to S5

Suporting Results and Discussion (Eyring analysis)

SI References

Materials and Methods

Eyring analysis. To obtain the activation parameters for the peroxygenase reaction of *Af*AA11B with $(GlcNAc)_4$, two forms of the Eyring equation were used (Equation (1) and Equation (2)):

$$\Delta G^{\#} = -RT\ln\left(\frac{k_{\mathrm{cat}}h}{k_{\mathrm{B}}T}\right) \qquad (1)$$

$$\ln\left(\frac{k_{\mathrm{cat}}}{T}\right) = \ln\left(\frac{k_{\mathrm{B}}}{h}\right) + \frac{\Delta \mathrm{S}^{\#}}{R} - \frac{\Delta \mathrm{H}^{\#}}{RT} \qquad (2)$$

where k_{cat} is the measured rate of the reaction, $\Delta G^{\#}$ is the change in activation free energy, $\Delta S^{\#}$ is the change in activation entropy, $\Delta H^{\#}$ is the change in activation enthalpy, h is Planck's constant, k_{B} is Boltzmann's constant, R is the gas constant, and T is the absolute temperature. The determined k_{cat} values were fitted to the linear form of the Eyring equation (2) using linear regression (ln k_{cat}/T vs. $1/T$) that was performed using OriginPro 2018 (OriginLab Corporation, Northampton, Massachusetts, USA). $\Delta H^{\#}$ was determined from the slope of the resulting straight line ($-\Delta \mathrm{H}^{\#}/R$). $-T\Delta S^{\#}$ was determined from the relationship described in Equation (3):

$$\Delta G^{\#} = \Delta H^{\#} - T\Delta S^{\#} \qquad (3)$$

To obtain the activation energy (E_{a}) and the frequency factor (A) of the reaction, the Arrhenius equation was used, Equation (4).

$$\ln k_{\mathrm{cat}} = \ln A - \frac{E_{\mathrm{a}}}{RT} \qquad (4)$$

The determined k_{cat} values were fitted to the linear form of the Arrhenius equation (4) and linear regression (ln k_{cat} vs. $1/T$) was performed using OriginPro 2018 (OriginLab Corporation, Northampton, Massachusetts, USA).

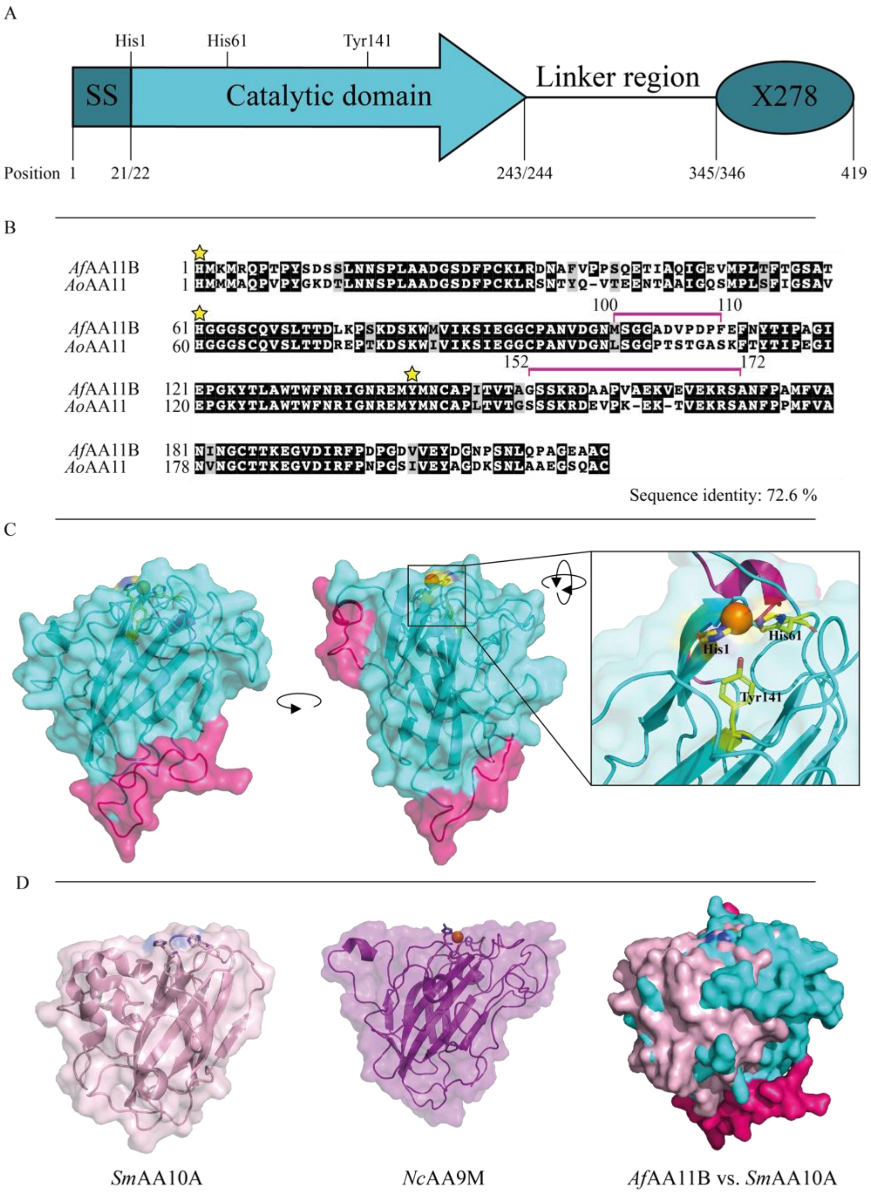

Figure S1. Overview of sequence and structure of *Af*AA11B and comparison with other LPMOs. (A) Domain structure of *Af*AA11B. The region connecting the catalytic domain and the X278 module comprises residues 244 to 345 and was assigned as a "linker" because it was predicted to be disordered by the PredictProtein server (https://predictprotein.org) server. (B) Sequence alignment of the catalytic domains of *Af*AA11B and *Ao*AA11[1] performed with the T-Coffee online server. Identical residues appear in black boxes, whereas similar residues appear in grey boxes. The two histidines (His1 and His61) and a tyrosine (Tyr141) that shape the copper site are marked with yellow stars. The pink brackets indicate the areas that are missing in the X-ray structure of *Ao*AA11. (C) Three-dimensional homology model in cartoon representation showing the immunoglobulin

like β-sheet core and the surface-exposed histidine brace. The model was generated using SWISSMODEL (https://swissmodel.expasy.org) with PDB-ID 4MAH as template. The pink areas, comprising residues 100-110 and 152-175, indicate regions where the model is less reliable, due to the template structure being incomplete. The image to the far right shows a close-up of the histidine brace with the copper coordinating histidines and the tyrosine in the axial position shown with yellow carbons. The copper atom is displayed as orange sphere. (D) Illustration of the flat surface topology typically found in LPMOs active on crystalline substrates illustrated by the X-ray structures of *Sm*AA10A (PDB: 2BEM; [2]) and *Nc*AA9M (PDB: 4EIS; [3]). All structures are shown in cartoon representation with a transparent surface (60%). The side chains of the N-terminal histidine, the second histidine in the active site and the aromatic amino acid in the proximal axial copper coordination position are shown as sticks. All structures are oriented identically with the N-terminal histidine facing to the left. The panel to the right shows a superposition of *Af*AA11B (blue) and *Sm*AA10A (pink), illustrating differences in surface topology near the active site. Protein images were prepared with PyMOL.

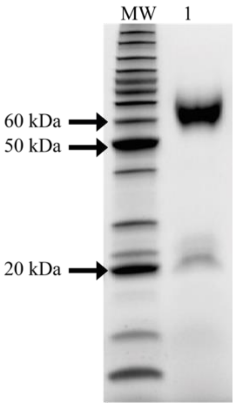

Figure S2. SDS-PAGE Gel of purified *Af*AA11B. Lanes: (MW) BenchMark^{TM} Protein Ladder (Thermo Fischer Scientific); (1) purified *Af*AA11B after three chromatographic purification steps where the final purification step displayed only one peak in the chromatograms suggesting that the fragments at 20 kDa are the result of degradation of *Af*AA11B during SDS-PAGE analysis.

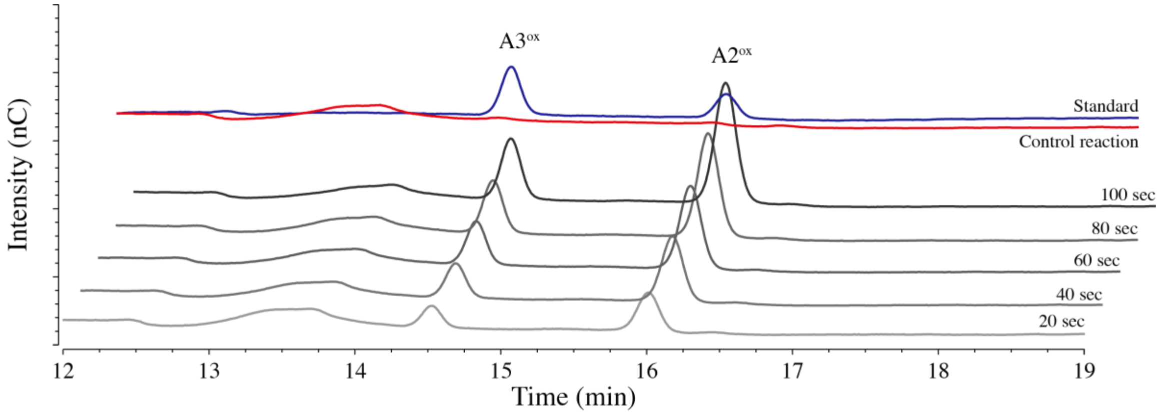

Figure S3. Typical HPAEC-PAD chromatograms showing product formation in reactions with *Af*AA11B and $(GlcNAc)_4$ and H_2O_2 (from the experiments determining K_m for H_2O_2 as the co-substrate). Reaction conditions: 0.1 µM enzyme, 250 µM H_2O_2, 2 mM $(GlcNAc)_4$ and 1 mM AscA) are shown as lines in gradations of black. Control reactions without AscA are shown as red lines. The blue chromatogram shows a standard containing 10 µM of the oxidized dimer and trimer.

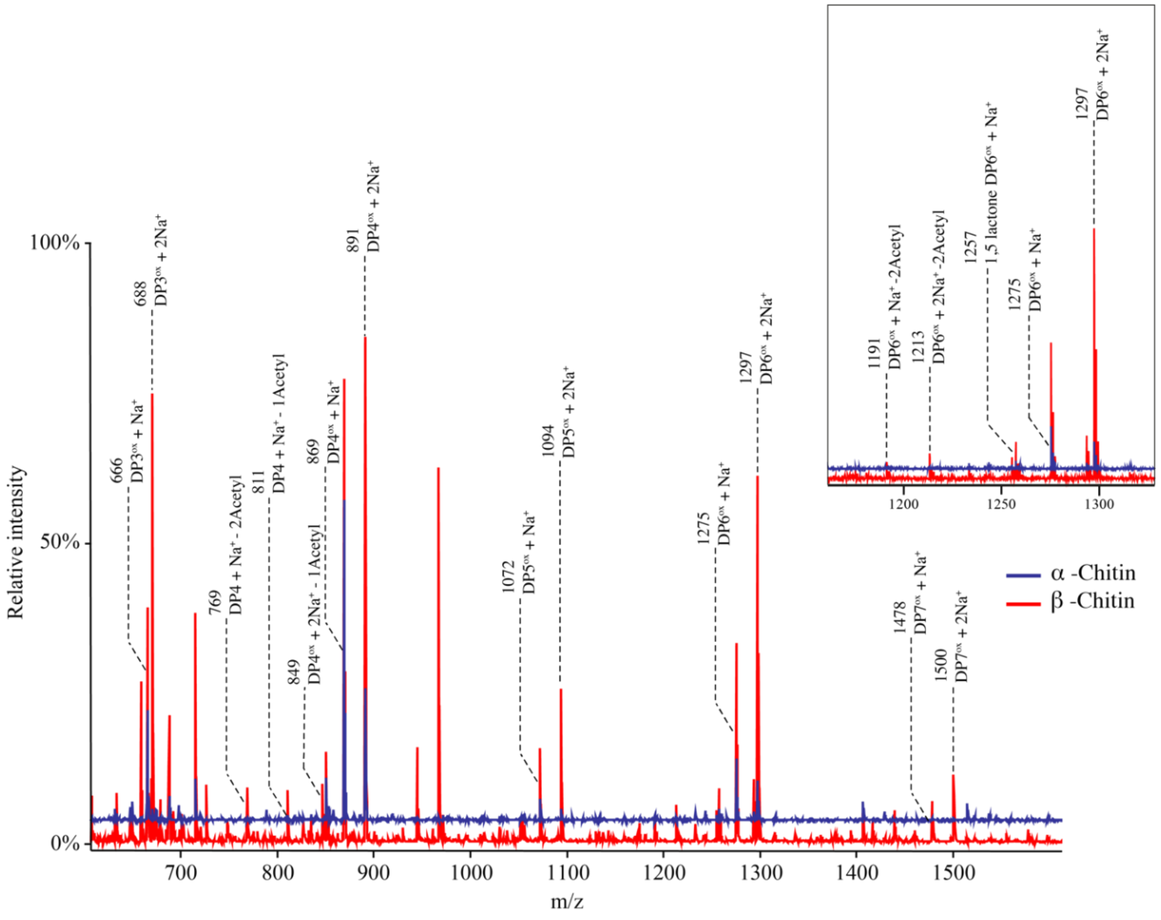

Figure S4. MALDI-ToF MS spectra showing oxidized products ranging from DP3 to DP7 (Δm/z 203) originating from α (blue) and β (red) chitin upon reaction with *Af*AA11B in the presence of 1 mM AscA and atmospheric oxygen levels. Labeled peaks represent sodium adducts of C1 oxidized products in the lactone form (Δm/z -2) and in the (dominating) aldonic acid form (Δm/z +16), as well as the sodium salt of the aldonic acid form (Δm/z +38). The insert to the right shows details for the DP6 cluster. The spectra also show small signals for partially de-acetylated native and oxidized products (Δm/z -42).

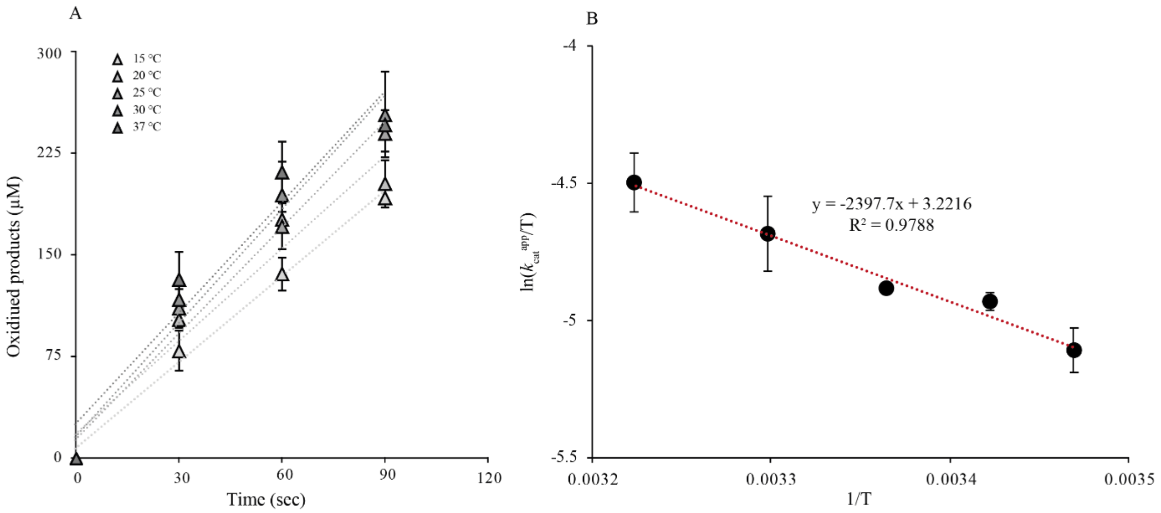

Figure S5. Temperature dependency and Eyring plot for the *Af*AA11B catalyzed oxidation of $(GlcNAc)_4$ in the presence of H_2O_2. (A) Linear time course experiments with 1 µM LPMO, 300 µM H_2O_2 and 1 mM AscA in the presence of 2 mM $(GlcNAc)_4$ at different temperatures (15-37 °C). (B) Eyring plot based on the calculated rate constants from (A).

Eyring analysis. Because *Af*AA11B catalyzes a strict peroxygenase reaction with a soluble substrate, it was, for the first time, possible to determine the activation parameters of the reaction by assessing the temperature dependency of the initial rates. The underlying time course experiments were performed with 1 µM LPMO in the presence of 300 µM H_2O_2, 1 mM AscA and 2 mM $(GlcNAc)_4$ at varying temperatures (15-37 °C). We could obtain linear progress curves for all reaction conditions, and the derived catalytic rates confirmed that the rate of catalysis is dependent on the temperature (Figure S5A). An Eyring plot based on the calculated rate constants (Figure S5B) gave a change in activation enthalpy ($\Delta H^{\#}$) of 4.7 ± 1.1 kcal/mol and a change in activation free energy ($\Delta G^{\#}$) of 17.3 ± 0.1 kcal/mol. The change in activation entropy ($\Delta S^{\#}$) was large and negative and the contribution of the entropic term ($-T\Delta S^{\#}$) was calculated to be 12.6 ± 1.2 kcal/mol. The Arrhenius analysis yielded an activation energy (E_a) of 5.5 ± 0.1 kcal/mol with a frequency factor (A) of $2.4 \cdot 10^4$ s^{-1}. Caution must be taken when the parameters are interpreted. Still, the large and negative contribution of the entropic term ($-T\Delta S^{\#}$ = 12.6 kcal/mol) to the activation free energy change suggests a high degree of order in the transition state.

SI References

(1) Hemsworth, G. R.; Henrissat, B.; Davies, G. J.; Walton, P. H. Discovery and characterization of anew family of lytic polysaccharide monooxygenases. *Nat. Chem. Biol.* **2014**, *10*, 122–126.

(2) Vaaje-Kolstad, G.; Houston, D. R.; Riemen, A. H. K.; Eijsink, V. G. H.; Van Aalten, D. M. F. Crystal structure and binding properties of the *Serratia marcescens* chitin-binding protein CBP21. *J. Biol. Chem.* **2005**, *280*, 11313–11319.

(3) Li, X.; Beeson IV, W. T.; Phillips, C. M.; Marletta, M. A.; Cate, J. H. D. Structural basis for substrate targeting and catalysis by fungal polysaccharide monooxygenases. *Structure* **2012**, *20*, 1051–1061.
